# Association of sTREM2 and YKL-40 With Alzheimer’s Disease Progression: A Systematic Review

**DOI:** 10.7759/cureus.111423

**Published:** 2026-06-24

**Authors:** Rasha Omer Babiker Mohamed, Abubaker M Elamin, Sara Salim Ali Ahmed, Salma B Mahmuod, Hounayda Abdelrahman Mohamed Hamid, Rawan Awad Amer Ahmed, May Medhat Aboaly

**Affiliations:** 1 Trauma and Orthopedics, Noble's Hospital, Douglas, IMN; 2 Internal Medicine, Ministry of Health, Shaqra, SAU; 3 Care of Elderly, Musgrove Park Hospital, Taunton, GBR; 4 Acute Medicine, Midland Metropolitan University Hospital, Birmingham, GBR; 5 Internal Medicine, Adhum General Hospital, Jeddah, SAU; 6 Anatomical Sciences, St George's University, St George's, GRD; 7 General Practice, Medical University of Lodz, Lodz, POL

**Keywords:** alzheimer's disease, amyloid, astrocyte, cerebrospinal fluid biomarkers, chi3l1, microglia, neuroinflammation, strem2, ykl-40

## Abstract

Neuroinflammation is recognized as a core feature of Alzheimer’s disease (AD). Soluble triggering receptor expressed on myeloid cells 2 (sTREM2) and chitinase-3-like protein 1 (YKL-40) are fluid biomarkers of microglial and astrocytic reactivity associated with AD progression. However, their coupling to amyloid and tau pathology, stage-specific roles, and prognostic utility remain unclear. This systematic review synthesized evidence from 2021 to 2025 on associations of sTREM2 and YKL-40 with AD progression. This review followed Preferred Reporting Items for Systematic Reviews and Meta-Analyses (PRISMA) 2020 guidelines. PubMed/Medical Literature Analysis and Retrieval System Online (MEDLINE), Cumulative Index to Nursing and Allied Health Literature (CINAHL), Institute of Electrical and Electronics Engineers (IEEE) Xplore, and Web of Science were searched (Jan 2021-Dec 2025), with citation searching. Duplicates were removed using EndNote X21 (Clarivate, London, UK). Two independent reviewers screened records using the Population, Intervention, Comparison, Outcomes, and Study Design (PICOS) criteria, with disagreements resolved by a third reviewer. Original human studies measuring sTREM2 and/or YKL-40 across the AD spectrum were included. Methodological quality was assessed using the Newcastle-Ottawa Scale (NOS). Due to heterogeneity in study design and outcomes, a narrative synthesis was performed. Thirteen studies were included: seven on sTREM2, five on YKL-40, and one on both. Six were low risk of bias, and seven were moderate. Cerebrospinal fluid (CSF) sTREM2 showed a biphasic pattern across disease stages, with early potential neuroprotective associations and later correlation with cortical atrophy and cognitive decline. A sex-APOE ε4 interaction was observed, with higher levels in female carriers. YKL-40 showed weak amyloid associations but strong coupling with tau pathology and neurodegeneration, influenced by vascular risk factors. Plasma YKL-40 predicted incident dementia and cognitive decline, and serum YKL-40 differentiated early dementia from controls with good diagnostic performance. sTREM2 and YKL-40 represent biologically distinct but complementary neuroinflammatory pathways in AD. sTREM2 reflects a stage-dependent microglial response, while YKL-40 reflects tau-associated astrocytic activation modulated by vascular factors. Longitudinal studies with concurrent biomarker assessment are needed to clarify their combined prognostic value.

## Introduction and background

Alzheimer's disease (AD) is a progressive neurodegenerative disorder and the leading cause of dementia worldwide, accounting for an estimated 60-70% of all dementia cases and affecting more than 55 million people globally, a figure projected to exceed 139 million by 2050 as populations age [1]. The pathological hallmarks of AD, extracellular amyloid-beta (Aβ) plaques and intracellular neurofibrillary tangles composed of hyperphosphorylated tau, have long dominated mechanistic and therapeutic frameworks for the disease [2]. However, it is now recognized that neuroinflammation constitutes a third core feature of AD pathogenesis, active early in the disease course and closely intertwined with proteinopathy progression [3]. Genome-wide association studies have identified numerous immune-related genetic loci as major risk determinants for late-onset AD, implicating both microglial and astrocytic biology, the two principal glial cell populations of the central nervous system, comprising the brain’s resident innate immune cells (microglia) and its most abundant homeostatic support cells (astrocytes), in disease susceptibility and trajectory [4,5]. These observations have catalyzed substantial interest in neuroinflammatory fluid biomarkers as tools for understanding disease mechanisms, staging pathological progression, and ultimately stratifying patients for emerging anti-inflammatory and glial-targeting therapeutic strategies. Despite this momentum, the precise roles and temporal dynamics of individual neuroinflammatory biomarkers across the AD continuum remain incompletely characterized, and their integration into clinical and research frameworks lags considerably behind that of established core biomarkers such as cerebrospinal fluid (CSF) Aβ42, phosphorylated tau, and total tau. These three core measures index amyloid, tau, and neurodegeneration, respectively, and constitute the basis of the amyloid-tau-neurodegeneration (ATN) classification framework, the biomarker-based system for staging AD biologically along these three pathological axes that is applied throughout this review.

Among the most studied neuroinflammatory biomarkers in AD are soluble triggering receptor expressed on myeloid cells 2 (sTREM2) and chitinase-3-like protein 1 (YKL-40, also known as CHI3L1) [6]. TREM2 is an innate immune receptor expressed selectively by microglia in the brain, and loss-of-function variants in the TREM2 gene confer a substantially elevated risk of late-onset AD, comparable in magnitude to the effect of a single copy of the APOE ε4 allele, the most common and strongest genetic risk factor for sporadic late-onset AD [7]. Upon activation, TREM2 undergoes proteolytic ectodomain shedding, releasing a soluble fragment, sTREM2, into the extracellular space and CSF, where it is measurable as an in vivo indicator of microglial reactivity [8]. YKL-40, by contrast, is an astrocyte-secreted glycoprotein whose CSF and plasma concentrations rise in response to neuroinflammation and astrocyte activation and whose levels have been reported to increase in the preclinical and prodromal stages of AD, correlate with tau pathology, and predict cognitive decline and progression to dementia [9]. A recent systematic review and meta-analysis by Yu et al. [10] quantified the pooled associations of sTREM2 and YKL-40 with markers of AD pathology. The present systematic review is distinct from that work in both scope and synthesis strategy. Rather than generating pooled effect estimates of biomarker-pathology associations, it focuses specifically on disease progression across the full clinical continuum, from cognitively unimpaired at-risk individuals through mild cognitive impairment (MCI) to established AD dementia, and deliberately adopts a stage-resolved narrative synthesis rather than meta-analytic pooling. This choice is motivated by the strongly stage-dependent and, in the case of sTREM2, biphasic behavior of these biomarkers, whereby collapsing their associations into a single summary estimate would average away precisely the neuroprotective-versus-neurotoxic and amyloid-versus-tau distinctions that this review seeks to characterize. The present synthesis is further confined to primary studies published between 2021 and 2025, capturing the most recent ATN-anchored cohort and trial evidence rather than the broader, predominantly earlier literature aggregated previously. Beyond this prior synthesis, the literature has been characterized by considerable heterogeneity in study design, patient populations, disease staging methods, and biomarker measurement platforms, yielding divergent and at times contradictory findings. The degree to which sTREM2 and YKL-40 reflect protective versus pathological neuroinflammatory states, their differential coupling to amyloid versus tau pathology, and their relative utility as prognostic markers across the AD spectrum are questions that have not been systematically evaluated in an up-to-date synthesis of the recent literature.

Given these knowledge gaps and the rapidly expanding evidence base generated by large multicenter cohort studies and biomarker-enriched clinical trials over the past five years, a rigorous and contemporary systematic review is warranted. The review was deliberately restricted to studies published between 2021 and 2025 to capture the most contemporary evidence base, in particular the large, multicenter cohorts and biomarker-enriched trials that have applied the ATN framework and standardized immunoassay platforms predominantly within the last five years, thereby maximizing methodological currency and cross-study comparability within the formal synthesis. Foundational earlier studies that first established the core biology and clinical relevance of sTREM2 and YKL-40, although predating this window and therefore not part of the formal synthesis, are drawn upon narratively in the Discussion to preserve mechanistic context. This timeframe thus balances the inclusion of the newest, most methodologically homogeneous primary evidence against continuity with the seminal biomarker literature that underpins its interpretation. The overarching aim of this systematic review is therefore to critically synthesize evidence published between 2021 and 2025 on the associations of sTREM2 and YKL-40 with AD progression, encompassing their relationships with amyloid and tau pathology, cognitive decline, and structural neurodegeneration across the clinical continuum from cognitively unimpaired at-risk individuals through to established AD dementia. By evaluating the breadth, consistency, and methodological rigor of the current evidence, this review seeks to delineate the pathobiological roles of both biomarkers, identify areas of convergence and divergence in the literature, and provide a foundation for their translational application in disease monitoring and therapeutic target engagement.

## Review

Methodology

Study Design

This systematic review was conducted and reported in accordance with the Preferred Reporting Items for Systematic Reviews and Meta-Analyses (PRISMA) 2020 guidelines [11].

Eligibility Criteria

Studies were eligible for inclusion if they met the following pre-specified criteria based on the Population, Intervention, Comparison, Outcomes, and Study Design (PICOS) framework [12]. With respect to population, eligible studies had to include human participants spanning any stage of the AD clinical spectrum, including cognitively unimpaired individuals with or without AD biomarker evidence, individuals with MCI, or patients with clinically or biomarker-confirmed AD dementia. Regarding the index exposure or biomarker, studies were required to measure sTREM2 and/or YKL-40 (CHI3L1) in any biological fluid matrix, including CSF, plasma, or serum. With respect to comparators, studies were required to include a reference or comparison group, such as cognitively unimpaired controls, an earlier disease stage, or an alternative ATN biomarker profile, to allow meaningful assessment of biomarker associations with AD progression. Eligible outcomes included any measure of AD pathological progression, such as amyloid-PET or CSF Aβ42 for amyloid burden, tau-PET or CSF phosphorylated tau and total tau for tau pathology, and MRI volumetrics, FDG-PET, or validated cognitive composites for neurodegeneration and cognitive decline. Concerning study design, only original research articles reporting primary data were eligible; review articles, editorials, opinion pieces, conference abstracts, letters, and case reports were excluded. Studies were restricted to those published in the English language between January 2021 and December 2025 to capture the most recent and methodologically contemporaneous evidence.

Information Sources and Search Strategy

A comprehensive and systematic literature search was conducted across four electronic databases: PubMed/Medical Literature Analysis and Retrieval System Online (MEDLINE), Cumulative Index to Nursing and Allied Health Literature (CINAHL), Institute of Electrical and Electronics Engineers (IEEE) Xplore, and Web of Science. The search was executed in Feb 2026, without restriction on the geographical location of study origin. The search strategy was constructed using a combination of Medical Subject Headings (MeSH) terms and free-text keywords relating to the biomarkers of interest and the target condition. The core search terms included: ("sTREM2" OR "soluble TREM2" OR "TREM2" OR "triggering receptor expressed on myeloid cells 2") OR ("YKL-40" OR "CHI3L1" OR "chitinase-3-like protein 1") AND ("Alzheimer's disease" OR "Alzheimer disease" OR "AD" OR "mild cognitive impairment" OR "MCI" OR "dementia" OR "cognitive decline" OR "neurodegeneration"). Search filters were applied to restrict results to publications from 2021 onwards and to exclude non-English language articles. In addition to the database search, manual citation searching of the reference lists of all full-text-eligible reports and selected relevant review articles was performed to identify any additional records not captured by the electronic search strategy. The full search strategy is available in Appendix 1.

Study Selection

All records retrieved from database searches and citation searching were imported into EndNote X21 reference management software (Clarivate, London, UK), which was used to identify and systematically remove duplicate records prior to screening. Following deduplication, the remaining records were independently screened by two reviewers in two sequential stages. In the first stage, titles and abstracts were evaluated for relevance to the review question, and records that were clearly irrelevant were excluded. In the second stage, full-text reports of all records that passed title and abstract screening were obtained and assessed in detail against the pre-specified eligibility criteria. Any disagreements between reviewers at either stage were resolved through discussion, with recourse to a third reviewer where consensus could not be reached. The reasons for exclusion at the full-text stage were recorded and are reported in the PRISMA flow diagram.

Data Extraction

A standardized, pre-piloted data extraction form was used to collect relevant information from each included study. Data extracted included: first author and year of publication; country of origin and cohort name; study design; participant population and diagnostic classification; sample size; mean age; biomarker(s) measured and biological matrix; AD staging framework or classification system applied; key findings relating to biomarker associations with amyloid pathology, tau pathology, cognitive outcomes, and neurodegeneration; and primary conclusions. Data extraction was performed independently by two reviewers, with discrepancies resolved by consensus. Where data were not explicitly reported but appeared derivable from the methods or results, the original authors were not contacted within the scope of this review.

Risk of Bias Assessment

The methodological quality of each included study was independently assessed by two reviewers using the Newcastle-Ottawa Scale (NOS) [13], a validated and widely recommended instrument for appraising the quality of non-randomized observational studies in systematic reviews. The cohort version of the NOS was applied to longitudinal and prospective cohort studies, and the adapted cross-sectional version of the NOS was applied to purely cross-sectional and case-control studies, as appropriate to each study's design. The NOS evaluates studies across three domains: the selection of study participants (maximum four stars), the comparability of study groups with adjustment for key confounders (maximum two stars), and the ascertainment of the outcome or exposure of interest (maximum three stars), yielding a maximum possible score of nine stars per study. For comparability, one star was awarded for adjustment for age, sex, and/or APOE ε4 genotype, the most critical confounders in AD biomarker research, and a second star for adjustment for at least one additional covariate such as education, comorbidities, or baseline cognitive status. Studies with scores of seven to nine stars were classified as low risk of bias, scores of four to six as moderate risk, and scores of zero to three as high risk. For cross-sectional studies, items pertaining to baseline outcome absence, follow-up duration, and follow-up completeness were scored as not applicable, consistent with established adaptations of the NOS for cross-sectional designs. Discrepancies in NOS scoring between reviewers were resolved by discussion.

Synthesis of Results

A formal meta-analytic synthesis was not conducted due to the substantial methodological and clinical heterogeneity across the included studies. This heterogeneity was multidimensional, encompassing variation in study design (cross-sectional, longitudinal, and mixed designs), patient populations and disease stages (ranging from cognitively unimpaired at-risk individuals to established AD dementia), biomarker measurement matrices (CSF, plasma, and serum) and assay platforms (enzyme-linked immunosorbent assay (ELISA), Meso Scale Discovery (MSD), and other immunoassay formats), the AD biomarker classification systems employed (including both clinical diagnostic criteria and the ATN framework), and the nature of the outcome measures reported (amyloid-PET, tau-PET, CSF core biomarkers, MRI volumetrics, and cognitive composites). The statistical approaches and the covariates included in multivariate models also differed substantially between studies. The aggregation of effect estimates under such conditions would risk producing a misleading pooled result that obscures meaningful biological variation across disease stages and study contexts and would violate assumptions of exchangeability required for valid meta-analytic inference. Findings were therefore synthesized using a structured narrative approach, with results organized thematically by biomarker and outcome domain to facilitate transparent interpretation of patterns of convergence and divergence across the included literature.

Results

Study Selection Process

**Figure 1:**
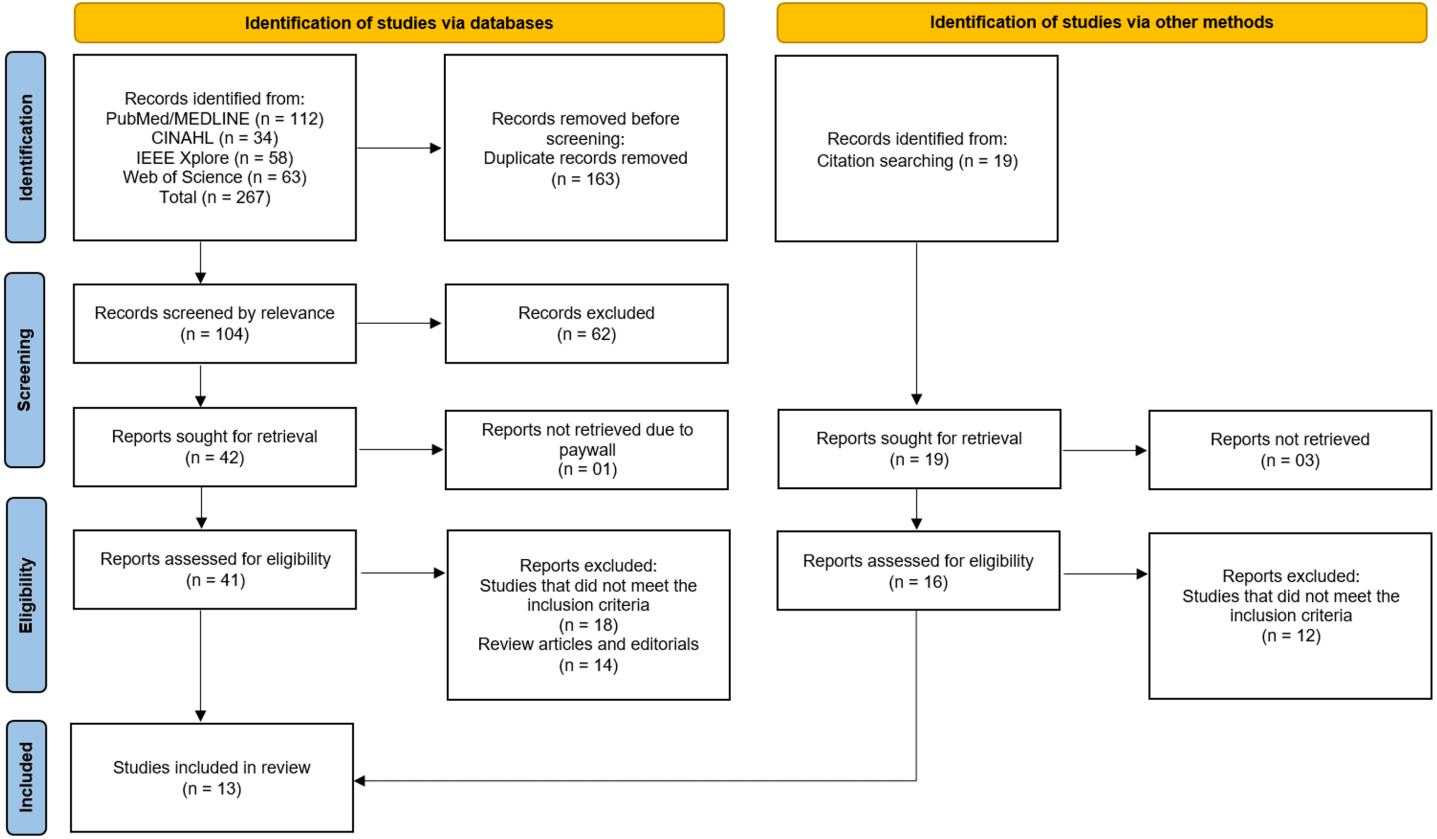
PRISMA flowchart illustrating studies' selection process PRISMA: Preferred Reporting Items for Systematic Reviews and Meta-Analyses; MEDLINE: Medical Literature Analysis and Retrieval System Online; CINAHL: Cumulative Index to Nursing and Allied Health Literature; IEEE: Institute of Electrical and Electronics Engineers

A systematic search of PubMed/MEDLINE (n = 112), CINAHL (n = 34), IEEE Xplore (n = 58), and Web of Science (n = 63) yielded 267 database records, supplemented by 19 records from citation searching. Following the removal of 163 duplicates, 104 records were screened for relevance, and 62 were excluded. Of the remaining 61 reports sought for retrieval (42 from databases and 19 from citation searching), four could not be obtained (one due to a paywall and three from citation sources). Full-text eligibility assessment of 57 reports (41 database; 16 citation-sourced) resulted in exclusion of 44 records: 30 did not meet inclusion criteria, and 14 were review articles or editorials. This yielded a final set of 13 original studies included in the review [14-26]. The complete selection process is illustrated in the PRISMA flow diagram (Figure 1).

Study Characteristics

Thirteen original studies published between 2021 and 2025 were included, collectively examining sTREM2, YKL-40, or both across the AD spectrum (Table 1). Seven studies focused exclusively on sTREM2 [14-20], five on YKL-40 alone [22-26], and one assessed both biomarkers concurrently [21]. Study designs comprised purely cross-sectional [15,17,20,22,23], mixed cross-sectional and longitudinal [14,16,21,24,26], and primarily longitudinal designs [18,19]. Sample sizes ranged from 80 [25] to 6,670 [24] participants. The majority used CSF as the primary biomarker matrix via validated immunoassay platforms, with two studies additionally examining peripheral blood matrices [24,25]. Studies were conducted across multiple countries and drew on established cohorts, including the Alzheimer's Disease Neuroimaging Initiative (ADNI) [15,16,18-20,26] and the ALzheimer's and FAmilies (ALFA+) study [21,23], with AD staging consistently performed using National Institute on Aging-Alzheimer's Association (NIA-AA) criteria and the ATN framework.

**Table 1:** Characteristics of included studies on sTREM2 and YKL-40 in Alzheimer's disease progression AD: Alzheimer's disease; ADNI: Alzheimer's Disease Neuroimaging Initiative; ALFA+: ALzheimer's and FAmilies study; Aβ: amyloid-beta; ATN: amyloid-tau-neurodegeneration; CSF: cerebrospinal fluid; CU: cognitively unimpaired; ELISA: enzyme-linked immunosorbent assay; FDG-PET: fluorodeoxyglucose positron emission tomography; fMRI: functional magnetic resonance imaging; GFAP: glial fibrillary acidic protein; ICAM1: intercellular adhesion molecule 1; MCI: mild cognitive impairment; MMSE: Mini-Mental State Examination; MRI: magnetic resonance imaging; MSD: Meso Scale Discovery; NfL: neurofilament light chain; NIA-AA: National Institute on Aging-Alzheimer's Association; NR: not reported; p-tau: phosphorylated tau; sTNFR1/2: soluble tumor necrosis factor receptors 1 and 2; sTREM2: soluble triggering receptor expressed on myeloid cells 2; t-tau: total tau; VCAM1: vascular cell adhesion molecule 1; YKL-40: chitinase-3-like protein 1

First author, year	Biomarker(s) studied	Country/cohort	Study design	Population	Sample size (n)	Mean age (years)	Biomarker matrix	AD staging/classification
Biel et al. [14] (2023)	sTREM2	Germany / Multi-centre (PREVENT-AD, TRIAD)	Cross-sectional + longitudinal	CU and MCI across early and late Aβ accumulation stages	402	NR	CSF sTREM2; amyloid-PET (centiloid); tau-PET; FDG-PET	Stratified by Aβ CSF+/PET− (early) vs Aβ CSF+/PET+ (late)
Li et al. [15] (2021)	sTREM2	China / ADNI	Cross-sectional	CU, MCI, AD dementia	1,035	~72	CSF sTREM2; CSF Aβ, p-tau, t-tau; neuroimaging	A/T profile severity groups; NIA-AA criteria
Nabizadeh [16] (2023)	sTREM2	Iran / ADNI	Cross-sectional + longitudinal tau-PET	Non-demented (Aβ− and Aβ+)	178	~73	CSF sTREM2; tau-PET; resting-state fMRI connectomes	Aβ-negative vs Aβ-positive (CSF Aβ42)
Woo et al. [17] (2023)	sTREM2	Canada / TRIAD (McGill)	Cross-sectional	CU, MCI, AD dementia	~300	NR	CSF sTREM2; plasma/CSF p-tau217, p-tau181, p-tau231; plasma GFAP; CSF 14-3-3 ζ/δ	ATN framework; tau-PET staging
Nabizadeh et al. [18] (2024)	sTREM2	Iran / ADNI	Longitudinal	CU, MCI, AD dementia	~400	NR	CSF sTREM2; amyloid-PET; tau-PET; FDG-PET; MRI	ATN classification (A/T/N framework)
Wang et al. [19] (2024)	sTREM2	China / ADNI	Longitudinal (growth curve model)	CU, MCI, AD dementia	1,017	~73	CSF sTREM2 (MSD platform)	ATN framework; NIA-AA 2018 criteria
Crook et al. [20] (2024)	sTREM2	UK / ADNI	Cross-sectional, ATN-stratified	CU controls, MCI (Aβ±/tau±), AD dementia	323	NR	CSF sTREM2; CSF p-tau181, Aβ42; amyloid-PET (18F-AV45); MRI	Aβ+/tau+, Aβ+/tau−, Aβ−/tau− subgroups
Warmenhoven et al. [21] (2024)	sTREM2 + YKL-40	Spain / ALFA+	Longitudinal (mean follow-up 3.28 years)	CU at risk of AD (family history)	353	60.9	CSF sTREM2, CSF YKL-40, CSF GFAP, plasma GFAP	Aβ status (CSF Aβ42/40 ratio)
Ferrari-Souza et al. [22] (2022)	YKL-40	Canada / TRIAD (McGill)	Cross-sectional	CU, MCI, AD dementia	121	NR	CSF YKL-40, CSF GFAP; amyloid-PET (18F-AZD4694); tau-PET (18F-MK6240)	Clinical spectrum (CU to AD dementia)
Pelkmans et al. [23] (2024)	YKL-40	Spain / ALFA+	Cross-sectional; structural equation modeling (path analysis)	CU at risk of AD (family history)	384	~61	CSF YKL-40, plasma GFAP, CSF GFAP; amyloid-PET; CSF p-tau181; NfL	Preclinical AD; Aβ status
Pase et al. [24] (2024)	YKL-40	USA, Iceland / AGES-RS, ARIC, CARDIA, Framingham Heart Study	Cross-sectional + prospective (up to 7.7 years)	Community-based dementia-free adults	6,558-6,670	NR (range ~45-85)	Plasma YKL-40 (MSD platform); brain MRI; CSF; cognitive composites	Incident dementia (clinical); MRI volumetrics
Wilczyńska et al. [25] (2021)	YKL-40	Poland / Single-centre	Cross-sectional, case-control	AD, vascular dementia, mixed dementia, CU controls (aged ≥60)	80	NR	Serum YKL-40, Aβ40, Aβ42, t-tau (ELISA)	Clinical diagnosis; MMSE severity
Wang et al. [26] (2025)	YKL-40	Multi-centre / ADNI	Cross-sectional + longitudinal	CU, MCI (Aβ±), AD dementia	~300+	NR	CSF YKL-40; CSF p-tau, t-tau, Aβ42; vascular neuroinflammatory markers (ICAM1, VCAM1, sTNFR1/2); MRI	ATN framework; A+MCI and T+ subgroups

sTREM2 Findings

CSF sTREM2 was consistently elevated in association with advancing AD pathology across all eight sTREM2 studies, though relationships proved stage-dependent. Li et al. [15] (n = 1,035) reported significant positive associations between CSF sTREM2 and both amyloid and tau markers (p < 0.001), while Wang et al. [19] (n = 1,017) confirmed that sTREM2 increased significantly with age over time (p < 0.0001). Biel et al. [14] demonstrated stage-specific amyloid-driven sTREM2 elevation in early Aβ-accumulators, where sTREM2 mediated longitudinal p-tau181 increases, a mediation absent in late accumulators. Nabizadeh conversely showed that higher sTREM2 attenuated tau-PET accumulation in Aβ-positive non-demented individuals, suggesting a protective microglial role at the amyloid stage [16]. Crook et al. (n = 323) revealed a biphasic pattern: neuroprotective sTREM2-tau correlations in MCI (r = 0.477-0.618), transitioning to negative associations with cortical volume and MMSE scores in established AD [20]. Woo et al. demonstrated that sTREM2 independently mediated early synaptic injury (14-3-3 ζ/δ) before hippocampal atrophy was visible [17]. Regarding cognition, sTREM2 showed no independent cognitive association in two studies [14,15], but Warmenhoven et al. [21] reported that higher baseline CSF sTREM2 predicted better longitudinal global cognition, memory, and executive function (Preclinical Alzheimer Cognitive Composite (PACC)) in 353 cognitively unimpaired at-risk individuals over 3.28 years. Wang et al. identified a significant sex-APOE ε4 interaction whereby female carriers showed higher sTREM2 than males (β = 0.146, p = 0.002) [19].

YKL-40 Findings

All six YKL-40 studies consistently reported elevated YKL-40 in association with AD pathology, though associations differed markedly by AD pathological substrate. YKL-40 showed weak and largely non-significant associations with amyloid pathology across studies [22,23,26], while demonstrating robust associations with tau pathology. Ferrari-Souza et al. (n = 121) showed that CSF YKL-40 was significantly associated with tau-PET burden independently of GFAP and mediated downstream neurodegeneration and cognitive impairment [22]. Pelkmans et al. (n = 384) used structural equation modeling to demonstrate that CSF YKL-40 mediated the association between p-tau181 and subsequent neuronal damage indexed by NfL, positioning YKL-40 as a mid-to-late cascade mediator [23]. Wang et al. corroborated tau associations in ADNI, showing these were partially mediated by vascular neuroinflammatory markers (ICAM-1, VCAM-1, sTNFR1/2; 13-79%) and moderated by hypertension [26]. Regarding cognition and neurodegeneration, Pase et al. provided the strongest prognostic evidence across 6,558-6,670 participants in four community cohorts, reporting that higher plasma YKL-40 predicted smaller total brain and hippocampal volumes, greater white matter hyperintensity, worse cognition, and increased incident dementia over up to 7.7 years [24]. Wang et al. demonstrated CSF YKL-40 predicted longitudinal cognitive decline and brain atrophy, particularly in hypertensive participants [26]. Wilczyńska et al. reported serum YKL-40 distinguished early dementia from controls with 85% sensitivity and specificity [25]. Key findings from all included studies are summarized in Table 2.

**Table 2:** Key findings and main outcomes of included studies AD: Alzheimer's disease; Aβ: amyloid-beta; ATN: amyloid-tau-neurodegeneration; CSF: cerebrospinal fluid; CU: cognitively unimpaired; FDG-PET: fluorodeoxyglucose positron emission tomography; fMRI: functional magnetic resonance imaging; GFAP: glial fibrillary acidic protein; ICAM1: intercellular adhesion molecule 1; MCI: mild cognitive impairment; MMSE: Mini-Mental State Examination; NfL: neurofilament light chain; PACC: Preclinical Alzheimer Cognitive Composite; p-tau: phosphorylated tau; sTNFR1/2: soluble tumor necrosis factor receptors 1 and 2; sTREM2: soluble triggering receptor expressed on myeloid cells 2; t-tau: total tau; VCAM1: vascular cell adhesion molecule 1; YKL-40: chitinase-3-like protein 1; ↑: increased

First author, year	Biomarker(s)	Biomarker levels across AD stages	Association with amyloid pathology	Association with tau pathology	Association with cognition/neurodegeneration	Key conclusions
Biel et al. [14] (2023)	sTREM2	CSF sTREM2 ↑ in early Aβ-accumulators (Aβ CSF+/PET-) relative to controls	Higher amyloid-PET centiloid associated with cross-sectional and longitudinal CSF sTREM2 ↑ in early accumulators only	Higher CSF sTREM2 mediated amyloid-PET–related longitudinal p-tau181 ↑; mediation not observed in late accumulators	Higher CSF sTREM2 associated with FDG-PET hypermetabolism (inflammatory glucose consumption); not directly associated with cognitive scores	sTREM2-mediated microglial activation occurs in response to earliest Aβ fibrillization and may facilitate subsequent p-tau increases; protective effects may dominate in later stages
Li et al. [15] (2022)	sTREM2	CSF sTREM2 ↑ with age and higher A/T profile severity across CU, MCI, and AD dementia groups	Higher CSF Aβ levels significantly associated with higher sTREM2 (p < 0.001)	Higher CSF p-tau and t-tau significantly associated with higher sTREM2 (p < 0.001)	sTREM2 not correlated with cognitive status, clinical conversion, or neurodegeneration on neuroimaging at baseline or longitudinally	CSF sTREM2 reflects a non-specific microglial state jointly driven by age, amyloid, and tau; it does not independently predict cognitive decline
Nabizadeh [16] (2023)	sTREM2	CSF sTREM2 ↑ in Aβ-positive vs Aβ-negative non-demented participants	CSF sTREM2 positively correlated with Aβ status; no mediation effect on Aβ–p-tau link	Higher CSF sTREM2 associated with attenuated longitudinal tau-PET accumulation in Aβ+ participants; tau spreading through functional connectomes reduced	Reduced tau spreading across fMRI functional networks in Aβ+ individuals with higher sTREM2	TREM2-related microglial activation may attenuate tau aggregate accumulation and spreading in the presence of amyloid pathology, suggesting a protective role at the amyloid stage
Woo et al. [17] (2023)	sTREM2	CSF sTREM2 elevated at early tau-PET stages, before hippocampal atrophy	CSF sTREM2 significantly associated with reduced CSF Aβ42/40 ratio	CSF sTREM2 significantly correlated with plasma p-tau217, p-tau181, and p-tau231	sTREM2 independently mediated early synaptic injury (14-3-3 ζ/δ) at incipient tau stages; later synaptic damage additionally driven by astrogliosis	sTREM2 is a key mediator of early synaptic loss, independently of tau accumulation; early amyloid detection may prevent this sTREM2-driven synaptic damage
Nabizadeh et al. [18] (2024)	sTREM2	CSF sTREM2 ↑ longitudinally across A/T/N groups; levels track biomarker severity	Positive association with amyloid-PET burden across ATN categories	Positive association with tau-PET burden; pattern varies by ATN stage	Longitudinal changes in CSF sTREM2 co-vary with neuroimaging markers of amyloid and tau accumulation over time	CSF sTREM2 demonstrates stage-dependent longitudinal dynamics within the ATN framework, supporting its utility as a dynamic microglial activation biomarker
Wang et al. [19] (2024)	sTREM2	CSF sTREM2 ↑ with age over time (p < 0.0001) across the full analytical sample	Not directly assessed	Not directly assessed	No significant sex difference in sTREM2 overall; APOE ε4-carrier women showed significantly higher sTREM2 than men (β = 0.146, p = 0.002)	CSF sTREM2 trajectory increases with age; sex–genotype interactions (APOE ε4 × female) significantly modulate sTREM2 dynamics, with implications for stratified biomarker interpretation
Crook et al. [20] (2024)	sTREM2	CSF sTREM2 ↑ in Aβ+/tau+ MCI and AD vs Aβ-/tau- MCI	Positive correlation with Aβ42 in Aβ- controls and MCI; negative correlation in AD group	Strong positive correlation with p-tau and t-tau in MCI groups (Aβ-: r = 0.618 and 0.615; Aβ+: r = 0.507 and 0.477); no significant correlation in AD	sTREM2 negatively associated with cortical volume in MCI and AD; negatively correlated with MMSE in AD	sTREM2 shows a biphasic response: neuroprotective associations with tau in MCI (early), shifting to neurotoxic associations with cortical atrophy and cognitive decline in established AD
Warmenhoven et al. [21] (2024)	sTREM2 + YKL-40	Both biomarkers detectable in CU at-risk individuals; levels vary by Aβ status	CSF sTREM2 and YKL-40 positively associated with executive function in Aβ- individuals	Not directly assessed as primary outcome	Higher CSF sTREM2 associated with better longitudinal global cognition (PACC), memory, and executive function independently of AD pathology; higher plasma GFAP associated with attention decline	TREM2-mediated microglial response associates with better longitudinal cognitive performance in at-risk CU individuals; distinct glial biomarkers exert different cognitive domain effects
Ferrari-Souza et al. [22] (2022)	YKL-40	CSF YKL-40 ↑ across the clinical spectrum from CU to AD dementia	CSF YKL-40 not significantly associated with amyloid-PET (18F-AZD4694) burden	CSF YKL-40 significantly associated with tau-PET (18F-MK6240) burden; association independent of CSF/plasma GFAP	Reactive astrocyte biomarkers (YKL-40, GFAP) mediated the effect of Aβ and tau on neurodegeneration and cognitive impairment	CSF YKL-40 selectively reflects tau-driven astrocyte reactivity, distinct from GFAP which reflects amyloid-driven astrogliosis; supports two astrocyte biomarker signatures in AD
Pelkmans et al. [23] (2024)	YKL-40	CSF YKL-40 ↑ in preclinical AD; rises later in the cascade than plasma GFAP	CSF YKL-40 did not mediate the association between soluble and insoluble Aβ aggregates	CSF YKL-40 mediated the association between Aβ-induced p-tau181 and downstream neuronal damage (NfL); not amyloid-specific	YKL-40 released into CSF in mid-cascade, mediating p-tau–driven neurodegeneration; plasma GFAP mediates the earlier amyloid cascade	CSF YKL-40 is a stage-specific mediator in the AD pathogenic cascade, acting between p-tau and neurodegeneration; its timing of elevation positions it as a mid-to-late preclinical marker
Pase et al. [24] (2024)	YKL-40	Higher plasma YKL-40 associated with smaller brain volumes and worse cognition across 4 cohorts	Not directly assessed via PET; replicated in clinic-based cohort with CSF Aβ	Not directly assessed	Higher plasma YKL-40 associated with smaller total brain volume, smaller hippocampal volume, greater white matter hyperintensity volume, worse cognitive composite scores, and increased incident dementia risk (prospective)	Plasma YKL-40 is a promising non-specific prognostic biomarker for accelerated brain aging and dementia risk in community populations, including risk independent of AD-specific processes
Wilczyńska et al. [25] (2021)	YKL-40	Serum YKL-40 ↑ in AD, vascular, and mixed dementia vs CU controls	Serum Aβ40 and Aβ42 did not confirm diagnostic utility for AD in this sample	Serum YKL-40 and t-tau correlated significantly with each other and with MMSE-measured cognitive severity	Serum YKL-40 distinguished CU from mild dementia with 85% sensitivity and 85% specificity; correlated with severity of cognitive decline	Serum YKL-40 is a sensitive early dementia biomarker, with diagnostic performance superior to amyloid markers in serum; comorbidities may influence levels
Wang et al. [26] (2025)	YKL-40	CSF YKL-40 ↑ with p-tau and t-tau; lower entorhinal cortex volume at higher YKL-40 levels	No significant direct association with CSF Aβ42 in primary analyses	CSF YKL-40 significantly correlated with p-tau and t-tau; mediated by vascular neuroinflammatory markers (ICAM1, VCAM1, sTNFR1, sTNFR2) at 13–79% proportions; moderated by hypertension	CSF YKL-40 predicted longitudinal cognitive decline and brain atrophy (longitudinal MRI); hypertension moderated the YKL-40–tau association	Vascular neuroinflammation partially mediates the YKL-40–tau pathway; hypertension is a significant moderator, highlighting vascular–neuroinflammatory interactions in AD progression

Risk of Bias Assessment

Using the NOS, six studies were rated moderate risk of bias (NOS 7-9) [14,16,18,19,21,24,26] and seven low risk (NOS 4-6) (Table 3) [15,17,20,22,23,25]. No study was rated high risk. Wang et al. [19] and Pase et al. [24] achieved the maximum score of 9. The predominant sources of methodological concern were cross-sectional designs precluding temporal inference [15,17,20,22,23], use of research-enriched cohorts limiting generalizability [15,16,18-21,23], inconsistent adjustment for vascular comorbidities, and limited demographic diversity across most cohorts. These limitations warrant caution when interpreting key findings: cross-sectional designs preclude definitive conclusions about causality or temporal directionality, particularly regarding the neuroprotective versus neurotoxic roles of sTREM2 across disease stages, while the predominance of homogenous, highly selected cohorts may overestimate biomarker performance and limit applicability to diverse community populations. The inconsistent adjustment for vascular comorbidities is especially relevant given the demonstrated moderation of the YKL-40-tau relationship by hypertension [26], suggesting that unmeasured confounding may have influenced effect sizes in some studies. Collectively, these methodological concerns highlight the need for large, diverse, prospective studies with standardized covariate adjustment to confirm the prognostic utility of sTREM2 and YKL-40 across the broader AD population.

**Table 3:** Risk of bias assessment using the Newcastle-Ottawa Scale for all 13 included studies

First author, year	Selection (max 4★)				Comparability (max 2★)		Outcome / Exposure (max 3★)			Total (max 9)	Overall RoB
	S1: Cohort representativeness	S2: Control group selection	S3: Biomarker ascertainment	S4: Outcome absent at start	C1: Key confounder controlled (age, sex, APOE)	C2: Additional confounder controlled	O1: Outcome assessment / blinding	O2: Follow-up duration adequate	O3: Follow-up completeness		Low
Biel et al. [14] (2023)	★	★	★	★	★	★	★	★	☆	8	Low
Li et al. [15] (2022)	★	★	★	NA	★	★	★	NA	NA	6	Moderate
Nabizadeh [16] (2023)	★	★	★	NA	★	★	★	★	☆	7	Low
Woo et al. [17] (2023)	★	★	★	NA	★	★	★	NA	NA	6	Moderate
Nabizadeh et al. [18] (2024)	★	★	★	★	★	★	★	★	☆	8	Low
Wang et al. [19] (2024)	★	★	★	★	★	★	★	★	★	9	Low
Crook et al. [20] (2024)	★	★	★	NA	★	★	★	NA	NA	6	Moderate
Warmenhoven et al. [21] (2024)	★	★	★	★	★	★	★	★	☆	8	Low
Ferrari-Souza et al. [22] (2022)	★	★	★	NA	★	★	★	NA	NA	6	Moderate
Pelkmans et al. [23] (2024)	★	★	★	NA	★	★	★	NA	NA	6	Moderate
Pase et al. [24] (2024)	★	★	★	★	★	★	★	★	★	9	Low
Wilczyńska et al. [25] (2021)	★	★	★	NA	★	☆	★	NA	NA	5	Moderate
Wang et al. [26] (2025)	★	★	★	★	★	★	★	★	☆	8	Low

Discussion

This systematic review synthesizes evidence from 13 original studies published between 2021 and 2025 examining the associations of sTREM2 and YKL-40 with AD progression across the full clinical spectrum. The collective findings affirm that both biomarkers capture biologically distinct but mechanistically complementary dimensions of neuroinflammation, with sTREM2 reflecting microglial reactivity and YKL-40 reflecting astrocyte activation in response to AD-related proteinopathy. Critically, neither biomarker functions as a simple, unidirectional indicator of disease severity; rather, both exhibit context- and stage-dependent relationships with core AD pathological substrates, cognition, and neurodegeneration that are only beginning to be fully understood. The principal added value of the present review lies in advancing beyond the only prior synthesis on this theme, the meta-analysis by Yu et al., which pooled cross-sectional biomarker-pathology associations into single summary estimates [10]. By contrast, the present review resolves these associations across the disease continuum, distinguishes the biphasic microglial (sTREM2) and tau-coupled astrocytic (YKL-40) trajectories, and appraises the prognostic and translational implications that a pooled estimate cannot capture, thereby offering a stage-specific, mechanistically integrated account rather than an averaged measure of association.

The finding that CSF sTREM2 is significantly elevated in association with both amyloid and tau pathology across multiple diagnostic groups [14,15,18,20] is broadly consistent with prior work by Suárez-Calvet et al. [27], who first demonstrated that CSF sTREM2 is increased in the early symptomatic phase of AD and correlates with markers of neuronal injury, and with subsequent evidence that sTREM2 peaks during the MCI stage before declining in advanced dementia. The present review adds important temporal resolution to this picture: Biel et al. [14] showed that sTREM2 mediates the amyloid-to-tau pathological cascade specifically in early Aβ-accumulators, while this mediation is absent in those with established plaque burden, suggesting that the window during which sTREM2-associated microglial activation is mechanistically coupled to tau propagation is narrow and restricted to the earliest phases of fibrillar amyloid formation. This is an important mechanistic refinement, as it implies that therapeutic strategies targeting TREM2 signaling are likely to be most effective if deployed before amyloid-PET positivity is established.

One of the most conceptually important and clinically significant themes to emerge from this review is the biphasic nature of sTREM2-associated microglial activity across the AD trajectory. Evidence from Crook et al. [20] and Nabizadeh [16], taken together, paints a coherent picture: in the pre-dementia amyloid stage, higher sTREM2 correlates with attenuated tau accumulation and reduced tau network spreading, consistent with a neuroprotective microglial phenotype that phagocytoses amyloid and limits tau seeding. In contrast, by the time established AD dementia is present, higher sTREM2 associates with cortical atrophy and poorer Mini-Mental State Examination (MMSE) scores, consistent with a neurotoxic shift. This biphasic model was anticipated by Ewers et al., who demonstrated that higher CSF sTREM2 in cognitively impaired AD patients was associated with reduced cognitive and clinical decline over four years, positing that sTREM2-mediated microglial activation is initially protective in symptomatic AD [28]. The present review extends this concept earlier into the disease continuum and provides imaging-based evidence for its temporal boundaries, which prior work had not fully delineated. The divergence between studies reporting beneficial and deleterious sTREM2 effects, therefore, likely reflects genuine biological stage-specificity rather than mere methodological heterogeneity.

The cognitive associations of sTREM2 across included studies were mixed, which mirrors the broader literature. Warmenhoven et al. [21] provided some of the most compelling prospective evidence to date that higher CSF sTREM2 predicts better longitudinal cognitive outcomes in preclinical at-risk individuals, independently of amyloid burden, while Li et al. [15] found no cognitive correlates of sTREM2 across a much larger cross-sectional ADNI sample. These discrepancies are likely attributable to disease stage: the neuroprotective signal from sTREM2 may be most detectable in cognitively intact, high-risk individuals before overt pathological accumulation has begun, whereas in mixed samples that include established disease, the beneficial and harmful effects of sTREM2 may cancel out cross-sectionally. The sex-APOE ε4 interaction identified by Wang et al. [19], showing that female APOE ε4 carriers exhibit disproportionately elevated CSF sTREM2 relative to male carriers, is a noteworthy finding with implications for precision medicine, as it suggests that the microglial response to amyloid risk is itself genetically and hormonally modulated.

The YKL-40 findings from this review are, in several respects, more internally consistent than those for sTREM2 and collectively point to a well-defined pathobiological role. The convergent evidence from Ferrari-Souza et al. [22], Pelkmans et al. [23], and Wang et al. [26] establishes that CSF YKL-40 is selectively coupled to tau pathology rather than to amyloid burden, a dissociation that distinguishes YKL-40 from GFAP, which is more strongly associated with amyloid accumulation. This biomarker-pathology specificity was first hinted at in the BioFINDER cohort by Janelidze et al. [29], who showed that CSF YKL-40 elevations were particularly strong in amyloid-positive individuals and correlated with tau, cortical thinning, and subsequent cognitive deterioration, findings that the present review's included studies now confirm and mechanistically elaborate using PET-based amyloid and tau quantification. The structural equation modeling by Pelkmans et al. [23] provides the clearest mechanistic positioning yet for YKL-40 within the AD cascade: as a mid-stage astrocytic mediator operating downstream of tau phosphorylation and upstream of overt neurodegeneration, consistent with the seminal work of Craig-Schapiro et al. [30], who first proposed YKL-40 as a neuroinflammatory biomarker predictive of progression to AD dementia. Querol-Vilaseca et al. further demonstrated the tau-specific expression of YKL-40 in astrocytes at the histological level, corroborating the fluid biomarker dissociations now observed in vivo across this review's included studies [31].

The demonstration by Wang et al. that vascular neuroinflammatory mediators, specifically ICAM-1, VCAM-1, and sTNFR1/2, partially mediate the YKL-40-tau association and that hypertension significantly moderates this relationship represents a meaningful advance beyond prior conceptualizations of YKL-40 as a purely parenchymal astrocyte marker [26]. This finding positions vascular inflammation as an active intermediary in astrocyte-driven tau pathology, not merely a comorbid confound. This mechanistic intersection of cerebrovascular and neuroinflammatory biology in the context of YKL-40 is consistent with Janelidze et al.'s [29] original observation that YKL-40 co-elevated with cerebrovascular markers in early AD and has important implications for risk stratification: individuals with hypertension may experience accelerated astrocyte-mediated tau propagation that is partly measurable via CSF YKL-40. From a clinical standpoint, the large-scale prospective evidence from Pase et al., demonstrating that plasma YKL-40 predicts incident dementia, smaller brain volumes, and cognitive decline across four independent community cohorts, significantly strengthens the case for YKL-40 as a scalable, blood-accessible prognostic biomarker that could complement or partially substitute for CSF measurement in population-level screening contexts [24].

Viewed together, the complementary profiles of sTREM2 and YKL-40 across the AD continuum begin to describe a coherent neuroinflammatory landscape: microglial activation, as indexed by sTREM2, appears to precede and partly drive tau phosphorylation at the earliest amyloid stage, whereas astrocyte reactivity, as indexed by YKL-40, emerges later and mediates the link between tau pathology and downstream neurodegeneration. Only one study in this review directly compared both biomarkers in the same cohort, and that study, Warmenhoven et al., found that they predicted different cognitive domain outcomes in preclinical individuals, with sTREM2 more broadly associated with preserved global cognition and YKL-40 more specifically linked to executive function in amyloid-negative individuals [21]. This domain specificity, combined with the temporal dissociation in their cascade positioning, supports the proposal by Lananna et al. that YKL-40 plays an active pathological role in AD beyond being a biomarker, since genetic reduction of Chi3l1 in mouse models improved amyloid phagocytosis and slowed neuroinflammation, suggesting that targeting astrocytic YKL-40 could complement microglial-targeting strategies such as TREM2 agonism [32].

Limitations

Several limitations of this systematic review must be acknowledged. First, the majority of included studies employed cross-sectional or mixed designs, which preclude definitive conclusions about the temporal sequencing of sTREM2 and YKL-40 elevation relative to clinical disease onset; longitudinal studies with sufficiently long follow-up are needed to establish predictive validity and mechanistic directionality. Second, considerable heterogeneity in study design, sample composition, biomarker measurement platforms, and covariate adjustment strategies limited the feasibility of formal meta-analytic pooling, and findings are therefore synthesized narratively. Third, most cohorts were derived from research-enriched populations, notably ADNI and ALFA+, which are predominantly non-Hispanic White, highly educated, and recruited on the basis of family history or biomarker positivity; these characteristics substantially limit the generalizability of findings to the broader, more diverse population of individuals at risk of or living with AD. Fourth, only one study in this review directly examined both sTREM2 and YKL-40 within the same participants, making it impossible to draw firm conclusions about the relative timing, interaction, or additive diagnostic value of the two biomarkers from the current evidence base. Fifth, peripheral blood measurements of both biomarkers remain insufficiently standardized across platforms and cohorts, limiting the comparability of plasma and serum results with CSF data. Sixth, the exclusion of non-English studies and the potential for publication bias may have introduced selection bias, as relevant null or negative findings and non-English language research could have been systematically omitted. Finally, the search window of 2021-2025, while appropriate for a focused systematic review of recent evidence, means that foundational earlier studies informing our mechanistic understanding of these biomarkers are referenced only narratively in the discussion rather than forming part of the formal synthesis.

## Conclusions

sTREM2 and YKL-40 are biologically meaningful and clinically informative neuroinflammatory biomarkers across the AD spectrum, each capturing a distinct glial response that is differentially coupled to amyloid and tau pathology, cognitive decline, and neurodegeneration. sTREM2 reflects a stage-dependent microglial response that is neuroprotective in the preclinical and early MCI phases, attenuating tau spread and preserving cognitive function, but shifts toward a neurotoxic profile in established dementia. YKL-40 reflects tau-driven astrocyte reactivity that mediates mid-cascade neurodegeneration, predicts longitudinal cognitive decline and brain atrophy, and is further modulated by vascular risk factors, most notably hypertension. Together, these biomarkers offer a richer and more temporally resolved portrait of neuroinflammatory dynamics in AD than either can provide alone. Future research should prioritize longitudinal cohort studies that concurrently measure sTREM2 and YKL-40, include diverse populations, standardize peripheral biomarker measurement platforms, and evaluate whether these inflammatory signatures can predict response to emerging disease-modifying therapies, particularly those targeting microglial and astrocytic pathways. The integration of sTREM2 and YKL-40 into multimodal biomarker panels has the potential to refine disease staging, improve prognostic accuracy, and ultimately support personalized approaches to AD intervention.

## Appendices

Appendix 1 

**Table 4:** Search strategy for databases

Database	Search string
PubMed/MEDLINE	(("sTREM2"[Title/Abstract] OR "soluble TREM2"[Title/Abstract] OR "TREM2"[Title/Abstract] OR "triggering receptor expressed on myeloid cells 2"[Title/Abstract]) AND ("YKL-40"[Title/Abstract] OR "CHI3L1"[Title/Abstract] OR "chitinase-3-like protein 1"[Title/Abstract]) AND ("Alzheimer's disease"[Title/Abstract] OR "Alzheimer disease"[Title/Abstract] OR "AD"[Title/Abstract] OR "mild cognitive impairment"[Title/Abstract] OR "MCI"[Title/Abstract] OR "dementia"[Title/Abstract] OR "cognitive decline"[Title/Abstract] OR "neurodegeneration"[Title/Abstract])) AND ((y_2021[Date - Publication] : y_2025[Date - Publication])) AND (english[Filter])
CINAHL (EBSCOhost)	(TI ("sTREM2" OR "soluble TREM2" OR "TREM2" OR "triggering receptor expressed on myeloid cells 2") OR AB ("sTREM2" OR "soluble TREM2" OR "TREM2" OR "triggering receptor expressed on myeloid cells 2")) AND (TI ("YKL-40" OR "CHI3L1" OR "chitinase-3-like protein 1") OR AB ("YKL-40" OR "CHI3L1" OR "chitinase-3-like protein 1")) AND (TI ("Alzheimer's disease" OR "Alzheimer disease" OR "AD" OR "mild cognitive impairment" OR "MCI" OR "dementia" OR "cognitive decline" OR "neurodegeneration") OR AB ("Alzheimer's disease" OR "Alzheimer disease" OR "AD" OR "mild cognitive impairment" OR "MCI" OR "dementia" OR "cognitive decline" OR "neurodegeneration")) AND (DT 2021-2025) AND (LA English)
IEEE Xplore	("sTREM2" OR "soluble TREM2" OR "TREM2" OR "triggering receptor expressed on myeloid cells 2") AND ("YKL-40" OR "CHI3L1" OR "chitinase-3-like protein 1") AND ("Alzheimer's disease" OR "Alzheimer disease" OR "AD" OR "mild cognitive impairment" OR "MCI" OR "dementia" OR "cognitive decline" OR "neurodegeneration") Filters Applied: 2021-2025
Web of Science	TS=((("sTREM2" OR "soluble TREM2" OR "TREM2" OR "triggering receptor expressed on myeloid cells 2") AND ("YKL-40" OR "CHI3L1" OR "chitinase-3-like protein 1") AND ("Alzheimer's disease" OR "Alzheimer disease" OR "AD" OR "mild cognitive impairment" OR "MCI" OR "dementia" OR "cognitive decline" OR "neurodegeneration"))) AND PY=(2021-2025) AND LA=(English)
